# Reducing the risk of viral contamination during the coronavirus pandemic by using a protective curtain in the operating room

**DOI:** 10.1186/s13037-022-00332-x

**Published:** 2022-08-06

**Authors:** Parastoo Sadeghian, Yang Bi, Guangyu Cao, Sasan Sadrizadeh

**Affiliations:** 1 Department of Civil and Architectural Engineering, KTH Royal Institute of Technology, Brinellvägen 23, 10044 Stockholm, Sweden; 2 Department of Energy and Process Engineering, Norwegian University of Science and Technology, Trondheim, Norway

**Keywords:** COVID-19, Operating room, Computational fluid dynamics, Airborne infectious disease, Thermal injury, Protective curtain

## Abstract

**Background:**

Airborne transmission diseases can transfer long and short distances via sneezing, coughing, and breathing. These airborne repertory particles can convert to aerosol particles and travel with airflow. During the Coronavirus disease 2019 (COVID-19) pandemic, many surgeries have been delayed, increasing the demand for establishing a clean environment for both patient and surgical team in the operating room.

**Methods:**

This study aims to investigate the hypothesis of implementing a protective curtain to reduce the transmission of infectious contamination in the surgical microenvironment of an operating room. In this regard, the spread of an airborne transmission disease from the patient was evaluated, consequently, the exposure level of the surgical team. In the first part of this study, a mock surgical experiment was established in the operating room of an academic medical center in Norway. In the second part, the computational fluid dynamic technique was performed to investigate the spread of airborne infectious diseases. Furthermore, the field measurement was used to validate the numerical model and guarantee the accuracy of the applied numerical models.

**Results:**

The results showed that the airborne infectious agents reached the breathing zone of the surgeons. However, using a protective curtain to separate the microenvironment between the head and lower body of the patient resulted in a 75% reduction in the spread of the virus to the breathing zone of the surgeons. The experimental results showed a surface temperature of 40 ˚C, which was about a 20 ˚C increase in temperature, at the wound area using a high intensity of the LED surgical lamps. Consequently, this temperature increase can raise the patient's thermal injury risk.

**Conclusion:**

The novel method of using a protective curtain can increase the safety of the surgical team during the surgery with a COVID-19 patient in the operating room.

## Background

Airborne transmission diseases can spread via released droplets from the human respiratory system. These droplets transfer during speaking, breathing, sneezing, and coughing [1, 2]. Due to evaporation, these water droplets can have various size distributions [3]. Moreover, these airborne particles can be contaminated with agents of infectious diseases, including tuberculosis, SARS-CoV, and measles [4–6]. These airborne infectious diseases are highly contagious since they can transmit through short and long distances, besides transferring from a contaminated person due to direct contact [7, 8].

During the outbreak of Coronavirus disease 2019 (COVID-19), most surgeries have been postponed in spring 2020 [9]; consequently, surgical patients have received delayed treatments [10]. On the other hand, these delays caused patients to suffer for a longer time till delivering surgical treatments. The pandemic has brought new challenges to the operating room’s (OR) performance. As a result, a clean environment in the OR has not been important only for patient safety. Protection of the healthcare workers (HCW) has also been considered to avoid the surgical team's infection from a COVID-19 patient.

An increasing number of studies indicate that airborne transmission is the main method of the SARS-CoV-2 virus spread among the populations [6, 11, 12]. These studies showed that the SARS-CoV-2 virus could not only spread over short distances through behaviours such as sneezing, coughing, and talking that produce large aerosol particles, but also through breathing that generates small aerosols. Some studies have proposed performing the surgery for COVID-19 patients in the negative pressure OR. In this regard, the spread of the SARS-CoV-2 virus has been controlled from penetrating adjacent rooms by gaps in doors and windows [13–15]. Hill et al. [16] introduced a novel method called “Corona Curtain” to guarantee the safety of the emergency department staff during intubations of a COVID-19 patient. This novel concept used a plastic drape role to make the walls of an intubation tent. Although their proposed concept had successful results in the early stages, they lacked scientific data to evaluate the system’s effectiveness. Some research works have suggested using personal protective equipment (PPE) [17, 18] and aerosol boxes [19] to protect the surgical team. However, Yánez Benítez et al. [20] reported that using PPE significantly reduced the attendance of HCWs, and more than 80% of respondents reported an increase in surgical fatigue. Therefore, there is a high demand to propose new approaches to protect health care workers without compromising their performance.

The previous literature mainly proposed protective methods for HCW during the treatment of COVID-19 patients in the hospital wards. In contrast, this study investigated the safety of the medical team during the surgery with an infected patient in the OR. In this regard, the spread of airborne infectious diseases like COVID-19 from the surgical patient was studied. A new method was proposed to improve the safety of the surgical team. Moreover, the effect of various surgical lamps’ radiation intensity on temperature distribution in the wound area was experimentally and numerically evaluated.

## Methods

### Experimental study

#### Operating room laboratory

**Fig. 1:**
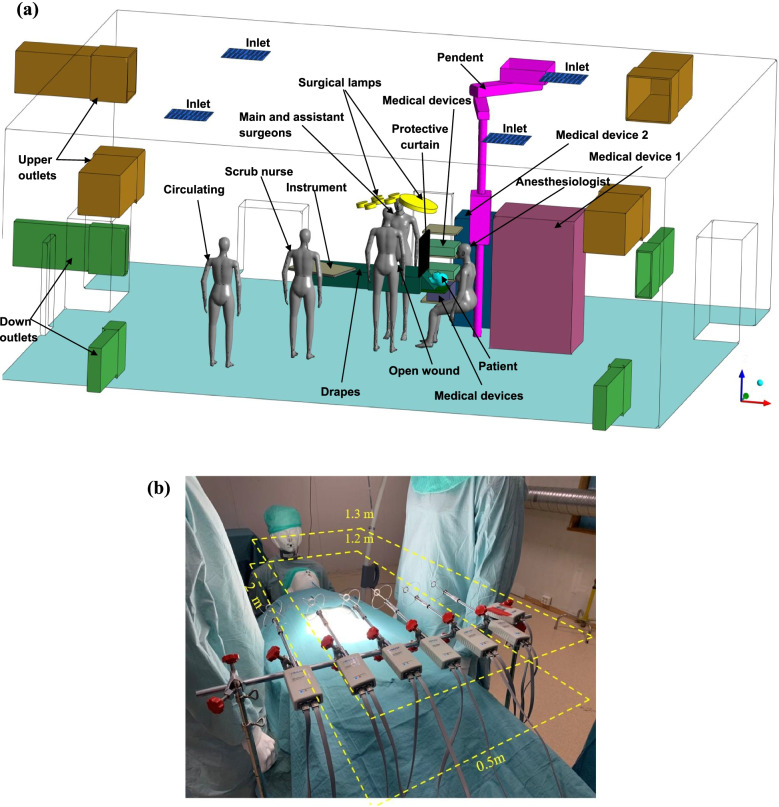
OR configuration with **a**) an isometric view, and **b**) measurement plane and instrument

All experimental measurements were finished in a full-scale OR laboratory of the Department of Energy and Process Engineering at Norwegian University of Science and Technology, Norway. The dimension of the laboratory is 8.73 m × 7.05 m × 3.25 m (length × width × height), and the total volume is 200m^3^. The OR was equipped with a mixing ventilation system with an air change rate of 20 ACH, as presented in Fig. 1. A variety of medical equipment was located in the OR, including an ultrasonic cleaner, two endoscope imagers, an anaesthesia machine, and two surgical ceiling pendants. All the equipment had a similar setup to a real OR at St. Olav hospital, Norway. The clean air supply rate was about 4000 m^3^/h, and the air extract rate was about 4242 m^3^/h, consequently resulting in a negative pressure condition of -5 Pa. The air temperature was 20℃ in the laboratory room. Two LED surgical lights were used; in which the first lamp was a Z 500 M lamp (made by the Meditech (India) company), and the second one was a STARLED3 NX lamp (manufactured by the ACEM Medical Company). The surgical lamps had a calorific value, and their heating power at the maximum brightness is shown in Table 1.

**Table 1:** Heating power of heat sources

Heat source	Heating power (W)
Surgical lamp 1	**61**
Surgical lamp 2	**74**
Supersonic cleaner	**45**
Endoscope imager	**232**
Main surgeon	**150**
Assistant surgeon	**150**
Scrub nurse	**140**
Circulating nurse	**140**

There were six thermal manikins in the laboratory room, including an anaesthesiologist nurse, a circulating nurse, a scrub nurse, a patient, a main, and an assistant surgeon. The skin surface temperature of the anaesthesiologist and patient was constant and regulated by a temperature control device. Their skin surfaces were set at 33℃ for the head, 30℃ for the arm, 31℃ for the chest, and 29℃ for the legs. Other thermal manikins generated a constant heat flux, as listed in Table 1. The heating power of each manikin was set according to the ASHRAE standard [21].

#### Experimental equipment and setup

AirDistSys5000 was used to measure the turbulence intensity, air velocity (with an accuracy of 0.2 m/s), and temperature (with an accuracy of 0.2℃) distributions. Overall, there were 66 measurement points, as presented in Fig. 1b. The applied instrument supported simultaneous measurements with up to eight instruments in series, of which six were used in this study. The instrument levels were placed according to the position shown in Fig. 1b, and the distance between the two instruments was 0.1 m, located 1.2 m in height above the floor. The whole measurement lasted for three minutes, and the data was recorded every two seconds, resulting in 90 data sets. The measurement was repeated after all devices were moved 20 cm horizontally along the operating table. Moreover, the power of all heat sources was measured with COLTECH EMT707CTL.

### Numerical study

Hospitals and operating room environments are complex, and conducting experimental studies to investigate the airflow field and contaminant levels might be difficult, expensive, and time-demanding.

Alternatively, the computational fluid dynamics (CFD) technique is powerful for predicting the airflow field and conducting high-quality parametric studies. In this regard, we prepared a replica model of the lab experiment to first validate our numerical model and then perform comprehensive numerical modelling and data visualization.

#### Computational fluid dynamics technique

CFD is a part of fluid mechanics knowledge that has been mainly applied for visualization of the temperature and airborne particle distributions, air movement, the impact of different types of ventilation systems, and contamination level in the air in enclosed environments [22–27]. This technique adopts numerical methods and models to investigate the fluid flow. Due to the complicated behaviour of the indoor airflow and airborne particles movements, using CFD simulation has been a common approach to predict airflow fields. CFD simulations present highly accurate results for the case studies that controlling the background factors is challenging, like ORs. The below four steps are required to apply the CFD technique.

##### Geometry

In the first step, the geometry of the laboratory room, including the configuration of the staff and medical equipment, was generated (Fig. 1a). Some degree of simplification was used for producing the geometry for the unimportant equipment located far from the operating table.

##### Mesh generation

The second step in CFD technique application is subdividing the computational geometry into cells, called mesh. The mesh resolution needs to have optimal value to save calculation time and computation resources. Finer mesh increases the accuracy of the simulation results. Thus, it requires to be fine enough to provide an accurate solution. It is important that the obtained simulation results be independent of mesh resolution. This study accomplished a mesh independence study to assure the results were independent of the generated mesh. The grid resolution of 8 million cells was used in this study to guarantee high-quality CFD results.

##### Solving methods

To compute airflow, airborne particle movement, and gas distribution in the physical model, it was necessary to define the required numerical models, equations, and boundary conditions. It was required to set up conditions to supply air diffusers, exhaust grills and gas release. The Navier–Stokes equation is the fundamental equation for the computation of the fluid momentum in most CFD simulations.

Since a large number of surgeries have been postponed during the COVID-19 pandemic, the spread of the SARS-CoV-2 virus from a COVID-19 patient was investigated. For modelling this spread, the SF6 gas was released from the patient's mouth and nose. This simulation was accomplished by solving the gas transport model and equations. All the equations for the simulation of BCPs and COVID -19 disperse were solved using commercial CFD code Fluent 19.2. Moreover, a novel approach, locating a protective shield curtain between the patient’s head and lower body, was introduced to moderate the COVID-19 disperse (Fig. 1a).

##### Post-processing results

The post-processing software was used to extract and visualize the CFD simulation results for the airflow field, particle, and gas distribution. This software improved the insight into the simulation results by generating velocity and velocity vector contour plots, airborne contamination, and gas dispersion contour plots, including the variation range of each studied variable.

### Model validation

**Fig. 2:**
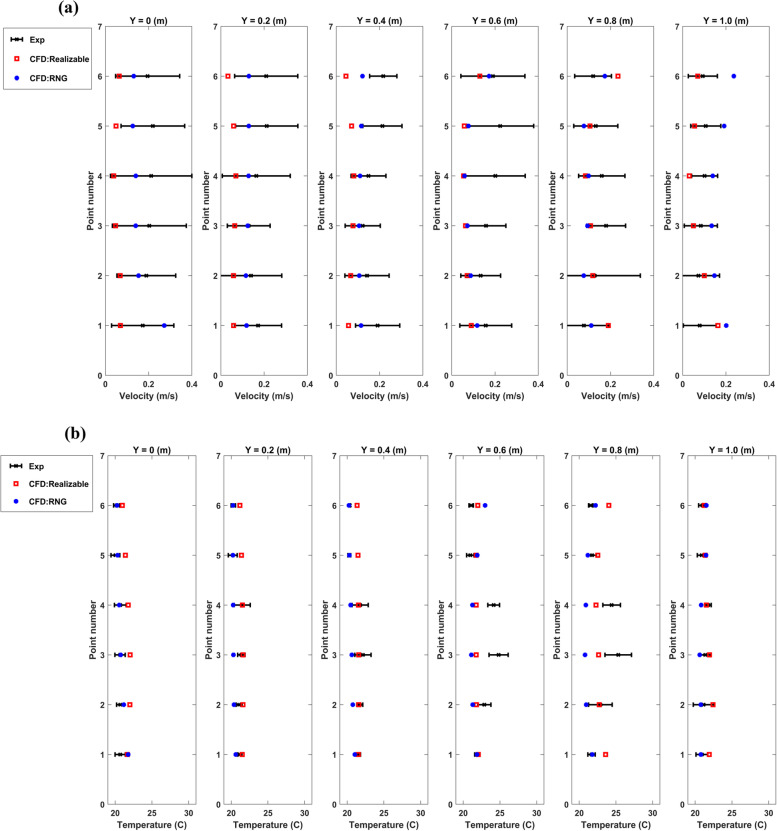
Comparison of **a**) velocity; and **b**) temperature distribution between CFD simulation and experimental results

The simulation results were compared with the experimental data to guarantee the applied CFD code precisely predicted the airflow behaviour in the OR. In this regard, the temperature and velocity distribution were validated at 66 measured points in the surgical microenvironment (Fig. 1b). Figure 2a compares two common turbulence models, RNG and Realizable k-ε models, with experimental results predicting the velocity distribution. The relative error between experimental and CFD simulation results was less than 5%. Thus, both turbulence models could successfully predict the airflow field.

During the experimental step, the temperature values above the surgical patient were registered since the heat generation from the medical equipment and manikins has an impact on the airflow behaviour. Furthermore, the temperature distribution was compared between the experimental and CFD simulation results, as presented in Fig. 2b. The maximum relative error was 10% in this comparison (Fig. 2b). Thus, the CFD simulation accurately predicts the airflow field and temperature distribution.

## Result

### Airflow behaviour

**Fig. 3:**
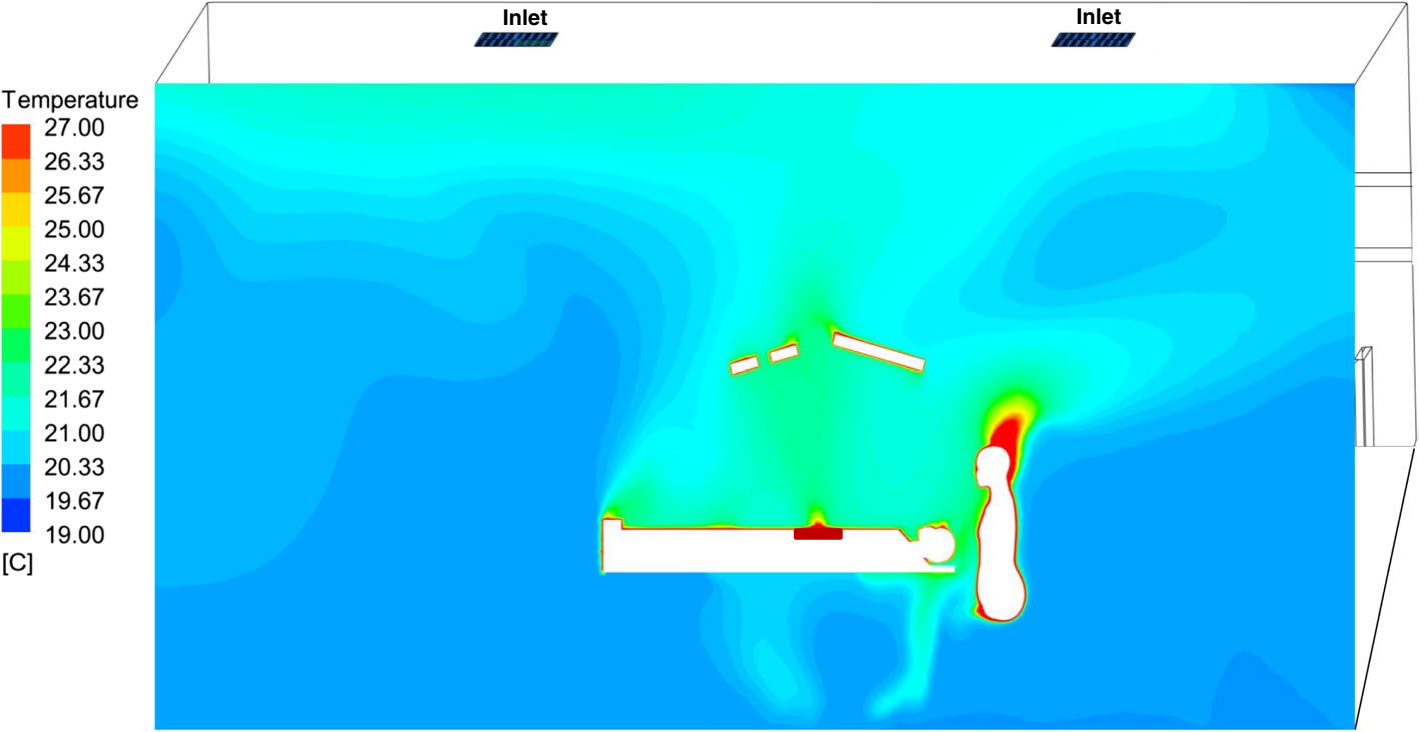
Visualizing the temperature distribution in the surgical microenvironment

The CFD simulation results showed that the air temperature above the surgical site was higher than in other areas. The maximum air temperature was 25℃ above the surgical site at the height of 1.2 m and 23.5℃ at 1.3 m above the floor (Fig. 3). However, the air temperature was around 21℃, further from the wound in the surgical microenvironment. A careful analysis of the measurement data confirmed this temperature variation in the surgical microenvironment. Furthermore, the surface temperature of the wound area increased from 20℃ to 40℃ during the experimental study.

**Fig. 4:**
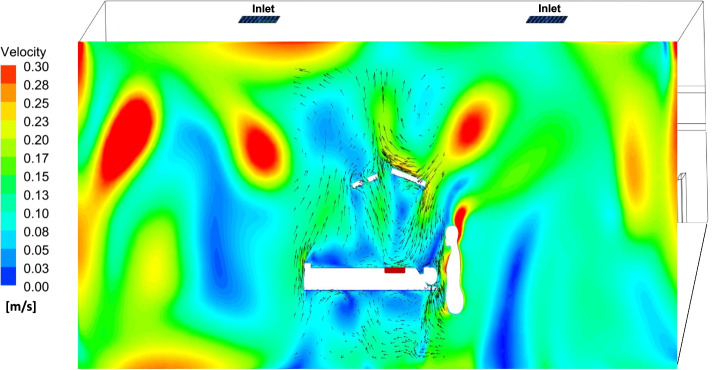
Visualizing the airflow behaviour in the surgical microenvironment

Figure 4 shows the velocity distribution and airflow behaviour in the surgical microenvironment above the patient.

### Contamination distribution

The SARS-CoV-2 virus was released from the patient during an ongoing surgery to evaluate the exposure level of the medical team. Overall, three different scenarios were numerically simulated to investigate the spread of airborne infectious diseases like the SARS-CoV-2 virus from the patient, as following:

- OR equipped with
-
  - Case 1: high intensity of the LED surgical lamp,
  - Case 2: low intensity of the LED surgical lamp, and
  - Case 3: high intensity of the LED surgical lamp with a protective curtain.

**Fig. 5:**
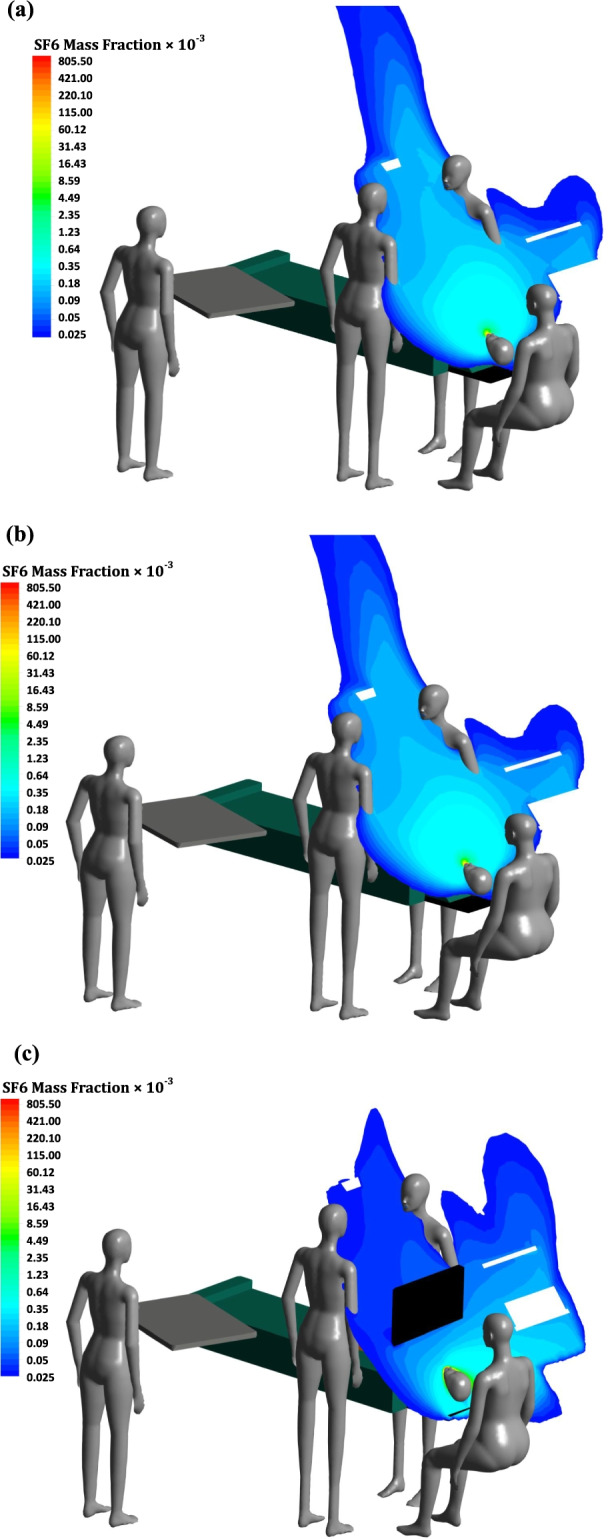
The spread of tracer gas from the patient's mouth **a**) case 1: high intensity-LED surgical lamp; **b**) case 2: low intensity-LED surgical lamp; and case 3: high intensity-LED surgical lamp with a curtain

The distribution of infectious airborne particles such as SARS-CoV-2 from the contaminated patient is presented for all cases in Fig. 5, in which SF6 tracer gas represents the airborne infectious particles. The results showed that in cases 1 and 2, the infectious airborne particles reached the maximum mass fraction of 0.18 × 10^–3^ at the breathing zone of the surgeon, and assistant surgeon, where located around the operating table (Fig. 5a, b). Using a protective curtain in Case 3 resulted in a 0.025 × 10^–3^ particle mass fraction in the surgeons’ breathing zone (Fig. 5c). In both cases, with and without protecting curtain, the virus was not obtained in the breathing zone of scrub and circulating nurses. However, the virus contamination was detected close to the anaesthesiologist nurse in all studied cases.

## Discussion

Several previous research studies reported burning damages in some patients’ wound areas by xenon, halogen, and shadowless surgical lamps [28, 29]. LED surgical lamps have been recognized as the best replacement for halogen lamps. However, the impact of various intensities of LED surgical lamps on wound thermal injury has not been clear. In the current study, the experimental results showed the wound surface temperature increased from 20℃ up to 40 ℃ by using a high intensity of the LED surgical lamp. This phenomenon might be due to radiation from the LED lamp, consequently heating the surface temperature of the wound. Thus, the high intensity of this surgical lamp has the potential to raise the wound temperature to 100% and cause severe thermal injury to the surgical patient. To prevent such injuries to the wound site, it is recommended to use low-intensity of LED lamps for long operations. Maximizing the distance between the surgical lamp and the wound site can substantially reduce the thermal radiation, consequently decreasing the risk of wound thermal injuries.

Since the outbreak of COVID-19, the safety of the medical team has become on priority. Various strategies have been proposed to increase the protection level of HCWs in hospitals. Hill et al. [16] suggested a novel concept entitled “Corona Curtain” that implemented plastic drape roles for making an intubation tent for a COVID-19 patient. Although their proposed concept was successful, the Corona Curtain was designed for hospital wards. Thus, it could compromise the performance of the surgical team in ORs. In this regard, the current study evaluated the exposure level of the surgical team while using a protective curtain located between the upper and lower patient’s body in the OR. The SF6 gas was released from the patient as representative of airborne infectious diseases spread like COVID-19. This contamination reached the breathing zone of surgeons in cases without the protective curtain (Cases 1 and 2). In this regard, the part of the surgical team close to the operating table needs a good protection level during surgery with COVID-19 patients. However, the CFD simulations showed that using this protective curtain reduced the exposure level up to 75% in the breathing area of the surgeons. Since using a protective curtain between the anaesthesiologist nurse and patient might affect the performance of this nurse, using a high protection mask rather than a surgical mask is highly recommended. Thus, using the proposed protective curtain reduces the spread of airborne infectious diseases from the patient and improves the safety of the surgeons.

## Conclusion

This study aimed to reduce the exposure level of the surgical team to airborne infectious contaminants during any future pandemic like COVID-19. In this regard, we proposed using a novel protective curtain to decrease the spread of SARS-CoV-2 from the patient to guarantee surgeons’ safety. Thus, using the proposed protection method could increase the safety of surgeons without compromising their performance. The SARS-CoV-2 distribution results showed that using a proper facemask is highly recommended for the anaesthesiologist who is close to the patient. The experimental and CFD simulation results highlighted the importance of avoiding using the LED surgical lamp at its high intensity to prevent thermal injuries. There is a demand for future studies to define the optimum light intensity, time duration, and distance from the LED surgical lamps.

## Abbreviations

CFD: Computational fluid dynamics
CFU: Colony-forming unit
COVID-19: Coronavirus disease 2019
HCW: Healthcare worker
OR: Operating room
PPE: Personal protective equipment
SSI: Surgical site infection
